# No difference in patient-reported outcome measures between private and public hospitals in the Netherlands: a cross-sectional analysis based on 170,150 hip and knee arthroplasties from the Dutch Arthroplasty Register

**DOI:** 10.2340/17453674.2026.45891

**Published:** 2026-05-09

**Authors:** Marije C VINK, Pelle BOS, Bart-Jan VAN DOOREN, Rinne M PETERS, Liza N VAN STEENBERGEN, Enrico DE VISSER, J Martijn BRINKMAN, B Willem SCHREURS, Wierd P ZIJLSTRA

**Affiliations:** 1 Department of Orthopedic Surgery, Frisius MC Leeuwarden; 2 Department of Orthopedic Surgery, University Medical Center Groningen; 3 Department of Orthopedic Surgery, Martini Hospital, Groningen, the Netherlands; 4 Department of Orthopedic Surgery, Flinders Medical Center, Adelaide, Australia; 5 Dutch Arthroplasty Register (LROI), ‘s Hertogenbosch; 6 Department of Orthopedic Surgery, Canisius Wilhelmina Hospital, Nijmegen; 7 Department of Orthopedic Surgery, Kliniek Orthoparc Rozendaal; 8 Department of Orthopedic Surgery, OrthoCare Clinics, Amersfoort; 9 Department of Orthopedic Surgery, Radboud University Medical Center, Nijmegen, the Netherlands

## Abstract

**Background and purpose:**

Private hospitals have become more frequent healthcare providers for arthroplasty surgery in the Netherlands. The aim of our study was to assess patient-reported outcome measures (PROMs) in patients who received primary total hip arthroplasty (THA), total knee arthroplasty (TKA), and unicompartmental knee arthroplasty (UKA) in private hospitals compared with patients from public hospitals, and to assess access to care based on mean travel distance to the healthcare facility.

**Methods:**

Patients operated on between 2014 and 2023 were included. Patient characteristics, preoperative, 3- or 6-, and 12 months postoperative PROMs (Numeric Pain Rating Scale [NRS] pain, EuroQoL 5-Dimensions [EQ-5D-5L], Hip disability and Osteoarthritis Outcome Score [HOOS-PS], Knee Injury and Osteoarthritis Outcome Score [KOOS-PS], Oxford Hip Score [OHS], and Oxford Knee Score [OKS]) were retrieved from the LROI. For analysis, repeated measurements were performed, using mixed-effect models adjusted for confounders. Primary endpoints for inference were NRS pain during activity, HOOS/KOOS-PS, and OHS/OKS at 3 months follow-up. Mean travel distance to the hospital was compared, as measure for access to care.

**Results:**

146,303 primary THAs, TKAs, and UKAs performed in public hospitals and 23,847 in private hospitals were included. Patients undergoing arthroplasty in private hospitals were generally younger and had a higher socioeconomic status and lower body mass index and American Society of Anesthesiologists Physical Status class. Both patients from private and public hospitals improved similarly and significantly after surgery. At 3-month follow-up, public hospitals showed marginal but statistically significant advantages in HOOS/KOOS-PS for THA, TKA, and UKA (mean differences 0.5 [95% confidence interval (CI) 0.1–0.9], 0.5 [CI 0.1–0.9], and 1.3 [CI 0.5–2.1], respectively). OKS favored public hospitals for TKA and UKA (–0.4 [CI –0.6 to –0.2] and –0.6 [CI –1.0 to –0.1]). NRS pain during activity favored private hospitals for TKA (–0.2 [CI –0.3 to –0.1]). All absolute differences were small and without statistical significance. Mean travel distance was significantly shorter for patients treated in public hospitals.

**Conclusion:**

There is no difference in PROMs between public and private hospitals after primary THA, TKA and UKA in the Netherlands. Based on mean travel distance, access to care was not compromised for high-risk patients.

In recent years, private hospitals have become more frequent healthcare providers in the Netherlands, especially for joint replacement surgery [1]. This growth has been driven by multiple factors, including extended insurance coverage by health insurance companies, and prolonged waiting lists in public hospitals following the COVID-19 pandemic [2]. In particular, total knee arthroplasty (TKA), unicompartmental knee arthroplasty (UKA), and total hip arthroplasty (THA) are increasingly performed in private hospitals [3]. To evaluate the patients’ opinion of success after arthroplasty, patient-reported outcome measures (PROMs) are used extensively [4].

The type of hospital in which the surgical procedure is performed may influence a patient’s perception of outcome. The Dutch healthcare system is based on mandatory health insurance for all residents, and patients may choose freely between public and private hospitals. Private hospitals tend to operate predominantly on younger and healthier patients [3]. Therefore, public hospitals perform increasingly more surgeries on older patients with more comorbidities. It raises questions about whether the market-driven healthcare system may inadvertently favor relatively healthier patients (American Society of Anesthesiologists Physical Status classification [ASA] I/II) in terms of ease of access compared with patients with more comorbidities (ASA III and higher). This potential bias could manifest in differences in waiting times, preoperative PROMs due to prolonged waiting lists, and variations in the completion of preoperative PROMs. Currently, there are no reports exploring distance and access to care alongside PROMs after arthroplasty in patients from private vs public hospitals in the Netherlands.

Our study aims to assess PROMs and access to care in patients undergoing primary THA, TKA, and UKA in a private hospital compared with patients from public hospitals, using data from the Dutch Arthroplasty Register (LROI).

## Method

### Study design and data source

This study is a cross-sectional study of all primary THAs, TKAs, and UKAs performed in the Netherlands between 2014 and January 1, 2023. The study cohort was identified by obtaining data from the LROI, a nationwide database of all arthroplasties performed in the Netherlands. Since 2007, information on patient, procedure, and prosthesis characteristics have been collected [1]. Currently, the LROI achieves a completion rate of over 97% for primary THA, TKA, and UKA [5]. For PROMs, data completeness was lower: in 2023, the proportion of patients who completed a preoperative and at least 1 postoperative PROM ranged from 34% to 42% [1]. The study is reported according to the STROBE guidelines [6].

### Data selection

We retrieved all patients who received a primary THA, TKA, and/or UKA for osteoarthritis (OA) between 2014 and 2023 in private and public hospitals, who had submitted a preoperative PROM and at least 1 postoperative PROM. In the Netherlands, the patient population of university hospitals differs strongly from those of private and public hospitals regarding pathology and comorbidity, due to centralization of high-complexity care to university hospitals. This population was considered non-representative, and procedures from university hospitals were therefore excluded from the analysis. PROMs are reported to the registry using a standardized questionnaire for all patients who underwent THA, TKA, and UKA preoperatively, and at 3 (for THA) or 6 months (for TKA and UKA) and 12 months postoperatively.

### Variables

Patient characteristics were obtained from the LROI, which included data on socioeconomic status (SES). The Dutch Institute of Social Research calculated SES scores based on 4-number postal codes using average income, percentage of inhabitants with low income, percentage of unemployed residents, and education levels [7]. These scores were divided into quintiles at the 20th, 40th, 60th, and 80th percentiles, and categorized into 3 groups: low SES (quintile 1), moderate SES (quintiles 2–4), and high SES (quintile 5). Hospital type was defined as “private” or “public.” Private hospitals are defined as specialized healthcare facilities and are usually smaller providers that often focus on 1 patient group, specialism, or treatment. Public hospitals are defined as healthcare facilities that are owned, operated, and funded by the government or a public entity.

Hip and knee arthroplasty-specific PROMs were collected. To evaluate health-related quality of life, pain levels, and functional outcomes, the Dutch Orthopedic Association has recommended a set of PROMs. For pain, this includes the Numeric Pain Rating Scale (NRS) for pain at rest and during activity. The NRS scoring system uses an 11-point Likert scale ranging from 0 (no pain) to 10 (severe pain). Health-related quality of life was measured using the EuroQoL 5-Dimensions (EQ-5D-5L) questionnaire, scoring from 0.0 (poor health) to 1.0 (perfect health) [8]. Physical functioning and disability after THA were assessed using the Oxford Hip Score (OHS), and the short version of the Hip disability and Osteoarthritis Outcome Score (HOOS-PS) respectively [9-11]. For TKA, the Oxford Knee Score (OKS) and the Knee Injury and Osteoarthritis Outcome Score (KOOS-PS) were used to assess these domains [11,12]. HOOS/KOOS-PS is measured on a scale from 0 to 100, lower scores indicating less disability (higher level of physical functioning). Scores of OHS/OKS range from 0-48, with lower scores indicating greater disability (lower level of physical functioning) [13].

### Outcomes

The primary outcomes selected for inference were differences in mean change of NRS for pain during activity, HOOS/OHS for hip patients and KOOS-PS/OKS for knee patients. These outcomes were prioritized because they directly reflect clinically meaningful recovery, impacting mobility, and daily function.

Secondary outcomes included differences in baseline PROMs between patients from private and public hospitals, as well as the mean travel distance to the healthcare facility. We consulted the literature for predefined minimal clinically important differences (MCIDs) [11,15-18], which were used to determine whether statistically significant differences in our results were likely to reflect clinically relevant changes. For THA, MCIDs of NRS pain, HOOS-PS, and OHS were 2.0 (95% confidence interval [CI] 1.8–2.2), 23 (CI 19–30), and 12.4 (CI 12.3–12.5), respectively. For TKA/UKA, MCIDs of NRS, KOOS-PS, and OKS were 1.5 (CI 1.3–1.7), 16 (CI 14–18), and 10.5 (CI 10.5–10.6), respectively.

### Statistics

Descriptive statistics on patients and PROM response rates are presented per arthroplasty according to type of hospital. Also, preoperative PROMs were described for both types of hospitals. Multivariable linear mixed-effect models were used to analyze PROMs in patients who submitted preoperative and at least 1 postoperative PROM to calculate the difference in progression between patients from different hospital types [14]. Because linear mixed-effects models use all available data, participants with incomplete follow-up were included under the assumption that data was missing at random. Travel distance was based on postal code of the patients’ home address and the hospital, according to the Google distance calculator. Mean travel distance per patient per province was calculated to estimate the accessibility to care. All models were adjusted for age, sex, body mass index [BMI], ASA class, and SES. The patient effect was considered a random effect in the model. The interaction of time by type of hospital in the models was considered to adjust for the within-subject variation over time. An unstructured covariance structure was used to model within-subject correlations over time, and parameters were estimated using restricted maximum likelihood (REML). Model assumptions were checked with residual plots for each dependent variable and were found to be acceptable. P values below 0.05 were considered statistically significant. Results were reported with 95% confidence intervals (CI). Statistical analyses were performed using IBM SPSS Statistics for Windows version 28.0 (IBM Corp, Armonk, NY, USA).

### Missing data handling

Missing data were handled using mixed-effects modeling under the Missing At Random (MAR) assumption. This approach incorporates all available data from participants, even if some follow-up measurements are missing, by estimating fixed and random effects based on observed information. As a result, the analysis provides unbiased estimates under the MAR assumption without requiring explicit imputation of missing values.

### Ethics, data sharing plan, funding, use of AI, and disclosures

Approval for this study was provided by the scientific advisory committee of the LROI. Ethical approval was not required according to the Dutch Medical Research Involving Human Subjects Act (WMO). The data used in this study was received completely anonymously, as part of routine clinical care, and complied with Dutch and EU data protection regulations. No funding was received for this study. No AI was used for this study. One author reported leadership roles as a potential conflict of interest (WPZ: president of Dutch Hip Society, Supervisory Board member of the Dutch Arthroplasty Register); all other authors reported no conflicts of interest. Complete disclosure of interest forms according to ICMJE are available on the article page, doi: 10.2340/17453674.2026.45891

## Results

**Figure 1:**
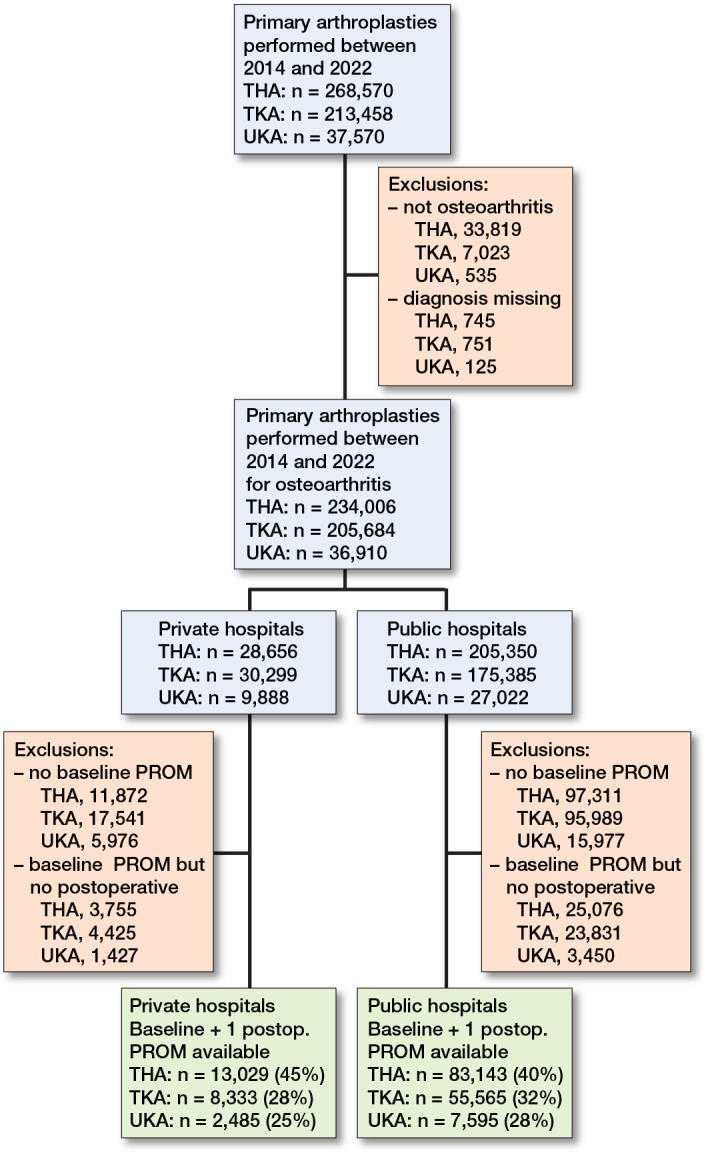
Flowchart of included procedures.

Between 2014 and 2023, a total of 519,598 THAs, TKAs, and UKAs in private and public hospitals were registered within the LROI. We retrieved all patients who received a primary THA, TKA, and/or UKA for osteoarthritis (OA) between 2014 and 2023 in private (n = 23,847) and public hospitals (n = 146,303), who had submitted a preoperative PROM and at least 1 postoperative PROM. A total of 170,150 procedures (96,172 THAs, 63,898 TKAs, and 10,080 UKAs) were included for analysis (Figure 1, Table 1). Response rates regarding PROMs of all patients varied per procedure (28–32% for TKA, 25–28% for UKA to 40–45% for THA) (Figure 1). Patients treated in private hospitals were generally younger, had a higher SES, lower BMI, and lower ASA class in all arthroplasty procedures examined (Table 1).

**Table 1:** Descriptive statistics of preoperative patient and procedure characteristics of all primary THA, TKA, and UKA performed in patients from private and public hospitals between 2014 and 2023 with at least a preoperative PROM and at 1 time point a postoperative PROM registered at 3 (hip), 6 (knee), or 12 months. Values are count (%)

Item	THA		TKA		UKA	
	Private	Public	Private	Public	Private	Public
	n = 13,029	n = 83,143	n = 8,333	n = 55,565	n = 2,485	n = 7,595
Age						
< 60	2,830 (22)	11,519 (14)	1,724 (21)	7,610 (14)	799 (32)	2,077 (27)
60–74	8,195 (63)	45,107 (54)	5,427 (65)	32,304 (58)	1,478 (60)	4,439 (58)
≥ 75	2,004 (15)	26,505 (32)	1,182 (14)	15,637 (28)	208 (8.4)	1,079 (14)
Sex						
Male	4,806 (37)	29,616 (36)	3,717 (45)	20,66 (37)	1,240 (50)	3,362 (44)
Female	8,223 (63)	53,502 (63)	4,616 (55)	34,882 (63)	1,245 (50)	4,233 (56)
ASA-class **^a^**						
I	4,542 (35)	12,263 (15)	2,558 (31)	5,267 (9.5)	922 (37)	1,105 (15)
II	8,376 (64)	53,102 (64)	5,697 (68)	36,584 (66)	1,523 (61)	5,25 (69)
III–IV	104 (0.8)	17,751 (21)	74 (0.9)	13,705 (25)	36 (1.5)	1,239 (16)
BMI **^a^**						
< 18.5	57 (0.4)	469 (0.6)	10 (0.1)	58 (0.2)	3 (0.1)	7 (0.2)
18.5–25	5,284 (41)	26,352 (32)	1,858 (22)	9,209 (17)	563 (23)	1,352 (18)
25–30	5,646 (43)	35,416 (43)	4,043 (49)	22,719 (41)	1,251 (50)	3,319 (44)
30–40	2,019 (15)	19,329 (23)	2,399 (29)	21,346 (39)	659 (27)	2,723 (36)
> 40	6 (0.1)	997 (1.2)	13 (0.2)	1,961 (3.5)	3 (0.1)	137 (1.8)
Socioeconomic status						
Low	1,607 (12)	14,867 (18)	1,081 (13)	10,027 (18)	308 (13)	1,164 (15)
Moderate	7,701 (60)	51,465 (63)	5,263 (64)	34,837 (63)	1,580 (64)	4,953 (66)
High	3,576 (28)	15,948 (19)	1,863 (23)	10,273 (19)	566 (23)	1,416 (19)
Previous operation						
Yes	166 (1.3)	1,179 (1.4)	3,016 (37)	13,961 (26)	719 (30)	1,660 (22)
No	12,613 (99)	81,748 (99)	5,042 (63)	40,573 (74)	1,690 (70)	5,755 (78)
Smoking						
Yes	1,058 (8.1)	7,261 (8.7)	629 (7.5)	3,960 (7.1)	213 (8.6)	662 (8.7)
No	11,963 (92)	74,929 (91)	7,699 (92)	51,411 (93)	2,271 (91)	6,923 (91)
Charnley						
A	5,526 (43)	35,481 (43)	3,896 (47)	22,253 (40)	1,343 (55)	3,914 (52)
B1	4,363 (34)	26,118 (32)	2,450 (30)	19,303 (35)	631 (26)	2,288 (30)
B2	2,278 (17)	19,056 (23)	1,466 (18)	12,001 (22)	390 (16)	1,243 (16)
C	787 (6.0)	2,048 (2.5)	446 (5.4)	1,767 (3.2)	85 (3.4)	114 (1.5)

a Significant difference, P <0.05.

THA: total hip arthroplasty, TKA: total knee arthroplasty, UKA: unicompartmental knee arthroplasty, PROM: patient-reported outcome measure, BMI: body mass index.

### PROMs

#### Baseline PROMs

Unadjusted mean baseline scores for THA, TKA, and UKA in all registered PROMs were significantly lower in patients from private hospitals (e.g., less pain and higher level of physical functioning) compared with patients from public hospitals (data not shown). However, after adjustment for confounders, only KOOS-PS of TKA patients showed a significantly lower baseline score for private hospitals. All other PROMs and arthroplasties did not show significant differences (Table 2).

**Table 2:** Adjusted mean difference of NRS pain during activity, HOOS-PS/KOOS-PS, and OHS/OKS preoperatively and 3- or 6-, and 12- months after total hip, total knee, and unicompartmental knee arthroplasty according to hospital type

PROM	Private hospital	Public hospital	Private vs public
	Mean and mean change (CI)	Mean and mean change (CI)	Difference in mean change (CI)
**Total hip arthroplasty**			
NRS Pain during activity			
Baseline	7.7 (7.6 to 7.8)	7.7 (7.6 to 7.8)	
Baseline to 3 months	–5.1 (–5.2 to –5.1)	–5.1 (–5.1 to –5.1)	–0.04 (–0.1 to 0.01)
Baseline to 12 months	–5.9 (–6.0 to –5.9)	–5.7 (–5.8 to –5.7)	–0.2 (–0.23 to –0.12) **^a^**
HOOS-PS			
Baseline	56.5 (55.7 to 57.2)	57.7 (57.0 to 58,4)	
Baseline to 3 months	–30.5 (–30.9 to –30.2)	–31.1 (–31.2 to –30.9)	0.5 (0.1 to 0.9) **^a^**
Baseline to 12 months	–35.9 (–36.3 to –35.6)	–35.4 (–35.6 to –35.3)	–0.5 (–0.9 to –0.1) **^a^**
OHS			
Baseline	17.8 (17.4 to 18.1)	17.0 (16.7 to 17.4)	
Baseline to 3 months	16.5 (16.4 to 16.7)	16.5 (16.4 to 16.6)	0.01 (–0.2 to 0.2)
Baseline to 12 months	19.7 (19.5 to 19.8)	19.2 (19.1 to 19.2)	0.5 (0.3 to 0.7) **^a^**
**Total knee arthroplasty**			
NRS Pain during activity			
Baseline	7.6 (7.5 to 7.7)	7.6 (7.6 to 7.7)	
Baseline to 6 months	–4.6 (–4.7 to –4.6)	– 4.4 (–4.5 to –4.4)	– 0.2 (–0.3 to –0.1) **^a^**
Baseline to 12 months	–5.2 (–5.3 to –5.1)	– 4.9 (–4.9 to –4.9)	– 0.3 (–0.4 to –0.2) **^a^**
KOOS-PS			
Baseline	55.0 (54.4 to 55.7)	56.9 (56.4 to 57.5)	
Baseline to 6 months	–20.0 (–20.4 to –19.7)	–20.5 (–20.7 to –20.4)	0.5 (0.1 to 0.9) **^a^**
Baseline to 12 months	–23.4 (–23.8 to –23.0)	–22.8 (–23.0 to –22.7)	– 0.6 (–1.0 to –0.1) **^a^**
OKS			
Baseline	19.2 (18.9 to 19.6)	18.7 (18.4 to 19.0)	
Baseline to 6 months	13.4 (13.2 to 13.6)	13.8 (13.7 to 13.9)	–0.4 (–0.6 to –0.2) **^a^**
Baseline to 12 months	15.8 (15.6 to 16.1)	15.4 (15.3 to 15.4)	0.5 (0.2 to 0.7) **^a^**
**Unicompartmental knee arthroplasty**			
NRS Pain during activity			
Baseline	7.4 (7.2 to 7.7)	7.5 (7.2 to 7.9)	
Baseline to 6 months	–5.0 (–5.1 to –4.9)	– 4.9 (–5.0 to –4.9)	– 0.1 (–0.2 to 0.03)
Baseline to 12 months	–5.5 (–5.3 to –5.6)	– 5.2 (–5.2 to –5.1)	– 0.3 (–0.4 to –0.1) **^a^**
KOOS-PS			
Baseline	52.0 (49.4 to 54.6)	54.2 (52.2 to 56.1)	
Baseline to 6 months	–20.7 (–21.4 to –20.0)	–21.9 (–22.3 to –21.5)	1.3 (0.5 to 2.1) **^a^**
Baseline to 12 months	–23.6 (–24.3 to –22.8)	–23.7 (–24.1 to –23.3)	0.1 (–0.7 to 0.9)
OKS			
Baseline	21.7 (20.4 to 23.1)	20.8 (19.8 to 21.8)	
Baseline to 6 months	14.7 (14.3 to 15.1)	15.3 (15.1 to 15.5)	– 0.6 (–1.0 to –0.1) **^a^**
Baseline to 12 months	16.3 (15.9 to 16.7)	16.2 (16.0 to 16.4)	–0.1 (–0.3 to 0.6)

a Significant difference.

CI: 95% confidence interval, NRS: Numeric Rating Scale, HOOS-PS: Hip disability and Osteoarthritis Outcome Score, KOOS-PS: Knee Injury and Osteoarthritis Outcome Score, OHS/OKS: Oxford Hip/Knee Score

#### Mean difference PROMs

**Figure 2:**
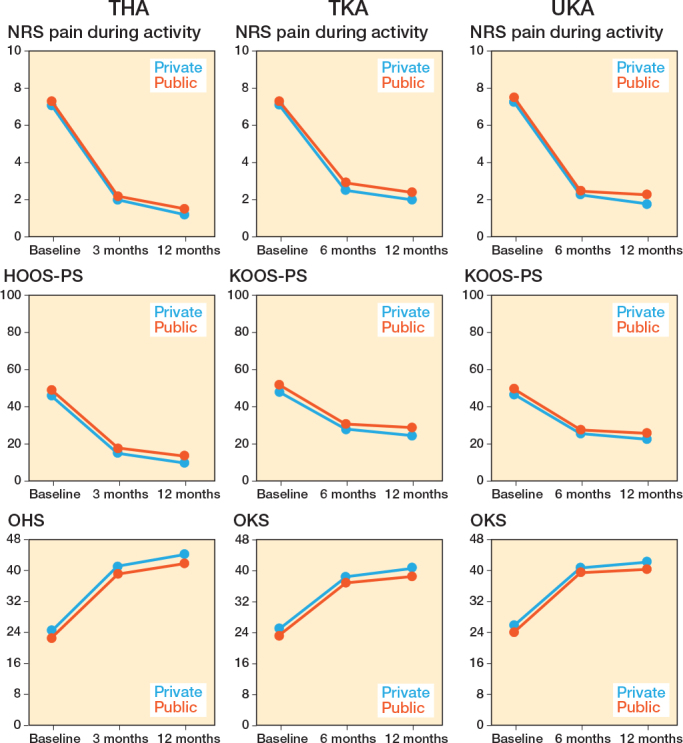
Mean changes in included primary total hip arthroplasties (THA), total knee arthroplasties (TKA), and unicondylar knee arthroplasties (UKA) for Numeric Rating Scale (NRS) pain during activity, Hip/Knee disability and Osteoarthritis Outcome Score (HOOS-PS/KOOS-PS), and Oxford Hip/Knee Score (OHS/KHS) preoperatively, 3- or 6-, and 12- months postoperatively for different hospital types.

Adjusted mean changes in patients from private and public hospitals improved significantly for THA, TKA, and UKA (Figure 2). Reported improvement in both patient groups exceeded minimal clinically important differences (MCIDs) in relation to pain during activity, OHS/OKS, HOOS-PS/KOOS-PS, as well as pain at rest, and EQ-5D.

Differences in mean change between patients from private and public hospitals demonstrated mixed results (e.g., some in favor of patients from private hospitals, some in favor of public hospitals, showing small absolute differences without clinical relevance; see Figure 2, Table 2, Table S3, Figure S3). At 3-month follow-up, public hospitals showed marginal but statistically significant advantages in HOOS/KOOS-PS for THA, TKA, and UKA (mean differences 0.5 [CI 0.1–0.9], 0.5 [CI 0.1–0.9], and 1.3 [CI 0.5–2.1], respectively). OKS favored public hospitals for TKA and UKA (–0.4 [CI –0.6 to –0.2] and –0.6 [CI –1.0 to –0.1]). NRS pain during activity favored private hospitals for TKA (–0.2 [CI –0.3 to –0.1]). All absolute differences were small, and no other PROMs differed significantly.

### Distance to healthcare facility

Overall, the mean travel distance to public hospitals was significantly shorter than to private hospitals (Table S4), although the absolute differences are small. The mean difference in travel distance was 24 km (CI 23.9–24.4) for THA, 24 km (CI 24.0–24.6) for TKA, and 19 km (CI 18.7–20.0) for UKA, with shorter distances to public hospitals.

## Discussion

The aim of our study was to assess PROMs in patients who received primary THA, TKA, and UKA in private hospitals compared with patients from public hospitals, using data from the LROI. Both groups improved significantly after arthroplasty, exceeding the MCIDs for the PROMs used, based on previous literature [11,15-18], suggesting that these changes are not only statistically significant but also clinically meaningful for patients, regardless of hospital type. The difference between hospital types was small and was not considered clinically relevant, being below the established MCID thresholds for all PROMs. Furthermore, the mean travel distance of patients treated in public hospitals was significantly shorter compared with patients from private hospitals, although absolute kilometer differences (19–24 km) were small and were therefore not considered to affect patients’ access to arthroplasty healthcare in the Netherlands.

Our findings are in line with other studies comparing patient satisfaction after arthroplasties. In a prospective cohort study by Adie et al., satisfaction after THA and TKA was analyzed, comparing patients from private hospitals with those from public hospitals in Australia [19]. They concluded that satisfaction with joint replacement was similar between patients from public and private hospitals. Patients from public hospitals had lower expectations preoperatively and may therefore be more likely to be satisfied after arthroplasty. In a survey 35 days post THA or TKA performed by Naylor et al. comparing patients from private hospitals with patients from public hospitals in Australia, the rate of satisfaction with the acute care for patients from private hospitals was not higher than for patients from public hospitals [20]. Nor were they more likely to recommend the hospital to others. The occurrence of any complication post-surgery appeared to be the most important predictor in perceived postoperative satisfaction.

In the Netherlands, private hospitals are required to follow guidelines provided by the Dutch healthcare inspectorate [21]. According to these guidelines, private hospitals are advised to refrain from treating patients classified as ASA III–IV. This restriction leads to a certain selection of patients directed to private hospitals. This selection is confirmed in our case mix, as most patients treated in private hospitals with THA, TKA, and UKA are between the age of 60 and 74 years, have an ASA class I–II and a BMI < 30. In addition, statistically significant differences in reported unadjusted baseline PROMs were observed. Patients in private hospitals reported less pain at rest and during activity, as well as higher functioning and quality of life preoperatively compared with patients from public hospitals. Differences in baseline PROMs are likely to be influenced by demographic factors, medical history, severity of condition, and general health situation. Patients choosing treatment in private hospitals may differ from patients treated in public hospitals in this regard. Furthermore, increasing waiting lists in public hospitals may have influenced baseline PROMs and are therefore explanatory factors in the results presented [2].

Regarding improvement in PROMs, we observed some differences, which probably depend on the effectiveness of the surgical intervention, quality of postoperative care, and willingness for rehabilitation. However, results varied per PROM and time point and did not point to a favorable type of hospital.

With the increasing number of private hospitals in the Netherlands, access to these facilities has become progressively more convenient for ASA I and II patients. This trend raises concerns about whether patients with higher perioperative risk profiles might encounter disparities in healthcare access, which could be translated into larger travel distances to public hospitals for ASA class 3 and 4 patients. However, our findings indicate that for THA, TKA, and UKA, the average travel distance was significantly shorter for public hospitals, although absolute differences were small (19–24 km).

Our previous study evaluating revision risk in private and public hospitals in the same study population [3] showed that patients treated in private hospitals were generally younger, and had lower ASA class and BMI, and higher SES compared with public hospital patients. This is in line with results from the present study, indicating that the population with completed PROMs is representative of the overall population. In our previous observational study, we concluded that patients treated in private hospitals had a lower risk of revision compared with patients treated in public hospitals for TKA, UKA, and THA, even after patient selection.

### Strengths

The main strength of this study is that it is based on a large sample of real-world registry data, which improves external generalizability.

### Limitations

We included all procedures in which at least a preoperative and 1 postoperative PROM had been registered, which could have resulted in selection bias [22]. Another limitation of our study is the high degree of missing PROM data: response rates were marginal, varying between 45% and 28% for patients who registered a preoperative as well as 1 postoperative PROM. This may lead to a non-response bias. We assumed that the data was missing at random, meaning that the likelihood of missing data depends on observed characteristics but not on unobserved outcomes. Nevertheless, nonresponse bias cannot be fully excluded, particularly if missingness is related to unobserved patient or provider factors, and this should be considered when interpreting our findings. Finally, patient populations differ between public and private hospitals, which compromises the ability to compare results between private and public hospitals. We adjusted for differences in case mix in our analyses, although there might be some residual confounding.

### Conclusion

Our study demonstrated that patients treated in both private and public hospitals improved significantly regarding PROMs after surgery, largely exceeding MCID thresholds. There was no clinically relevant difference in PROM change values between hospitals regarding hip and knee arthroplasty. The mean travel distance to public hospitals is shorter than to private hospitals, although absolute differences are small. Therefore, availability of arthroplasty care is currently not at risk for patients with higher ASA class in the Netherlands.

### Supplementary data

Tables S3–S4 and Figure S3 are available as supplementary data on the article page, doi: 10.2340/17453674.2026.45891

## Supplementary Material



## Notes

Authors contributed to: (1) study design and study protocol, (2) gathered data, (3) analyzed data, (4) initial draft, and (5) final draft. MCV, PB and BvD: (1), (2), (3), (4), (5); RMP and WPZ: (1), (2), (4), (5); LNvS, EdV, JMB and BWS: (1), (2), (5).

Handling co-editors: Keijo Makela and Robin Christensen

*Acta* thanks Lina Holm Ingelsrud and Richard N de Steiger for help with peer review of this manuscript.
