# Supplementary material for: No difference in patient-reported outcome measures between private and public hospitals in the Netherlands: a cross-sectional analysis based on 170,150 hip and knee arthroplasties from the Dutch Arthroplasty Register
Source: Acta Orthop. 2026 May 9;97:301–7. doi: 10.2340/17453674.2026.45891 (PMC13158729; doi:10.2340/17453674.2026.45891)
Supplement: Supplementary file 1 [file ActaO-97-45891-s1.pdf]

Supplementary data

**Table S3. Adjusted mean difference of NRS pain at rest, EQ-5D index score, and EQ-5D VAS preoperatively and 3- or 6-, and 12- months after total hip, total knee, and unicompartmental knee arthroplasty according to hospital type**			
**PROM**	Private hospital	Public hospital	Private vs public
	Mean and mean change (CI)	Mean and mean change (CI)	Difference in mean change (CI)
**Total hip arthroplasty**			
**NRS Pain at rest**			
Baseline	5.4 (5.3 to 5.5)	5.6 (5.5 to 5.7)	
Baseline to 3 months	–3.9 (–4.0 to –3.9)	–4.0 (–4.0 to –4.0)	0.1 (0.03 to 0.1) [a]
Baseline to 12 months	–4.3 (–4.4 to –4.3)	–4.3 (–4.4 to –4.3)	0.01 (–0.05 to 0.06)
**EQ-5D index score**			
Baseline	0.41 (0.40 to 0.42)	0.39 (0.39 to 0.40)	
Baseline to 3 months	0.29 (0.28 to 0.29)	0.28 (0.28 to 0.28)	0.01 (–0.00 to 0.01)
Baseline to 12 months	0.34 (0.33 to 0.34)	0.31 (0.31 to 0.32)	0.02 (0.02 to 0.03) [a]
**EQ-5D VAS**			
Baseline	54.1 (53.2 to 55.0)	53.9 (53.5 to 54.3)	
Baseline to 3 months	12.4 (12.0 to 12.8)	11.2 (11.0 to 11.4)	1.2 (0.8 to 1.6) [a]
Baseline to 12 months	14.3 (13.8 to 14.8)	11.6 (11.4 to 11.8)	2.7 (2.2 – 3.2) [a]
**Total knee arthroplasty**			
**NRS Pain at rest**			
Baseline	5.2 (5.1 to 5.3)	5.4 (5.3 to 5.5)	
Baseline to 6 months	–3.4 (–3.5 to –3.3)	–3.3 (–3.3 to –3.3)	– 0.1 (–0.2 to 0.1) [a]
Baseline to 12 months	–3.8 (–3.8 to –3.7)	–3.6 (–3.6 to –3.6)	– 0.2 (–0.2 to –0.1) [a]
**EQ-5D index score**			
Baseline	0.49 (0.48 to 0.5)	0.48 (0.47 to 0.48)	
Baseline to 6 months	0.23 (0.23 to 0.24)	0.22 (0.22 to 0.22)	0.01 (0.01 to 0.02) [a]
Baseline to 12 months	0.26 (0.26 to 0.27)	0.24 (0.24 to 0.24)	0.02 (0.02 to 0.03) [a]
**EQ-5D VAS**			
Baseline	60.3 (59.5 to 61.2)	60.0 (59.2 to 60.8)	
Baseline to 6 months	7.5 (7.0 to 8.0)	6.2 (6.0 to 6.4)	1.4 (0.8 to 1.9) [a]
Baseline to 12 months	9.1 (8.5 to 9.6)	6.7 (6.5 to 6.9)	2.4 (1.8 to 3.0) [a]
**Unicompartmental knee arthroplasty**			

NRS Pain at rest			
Baseline	5.1 (4.8 to 5.4)	5.0 (4.7 to 5.3)	
Baseline to 6 months	–3.8 (–3.9 to –3.7)	–3.7 (–3.7 to –3.6)	– 0.1 (–0.3 to 0.01)
Baseline to 12 months	–4.0 (–4.1 to –3.9)	–3.8 (–3.9 to –3.8)	– 0.2 (–0.3 to –0.03) [a]
**EQ-5D index score**			
Baseline	0.53 (0.51 to 0.56)	0.50 (0.48 to 0.52)	
Baseline to 6 months	0.25 (0.24 to 0.26)	0.27 (0.26 to 0.27)	–0.02 (–0.03 to –0.01) [a]
Baseline to 12 months	0.27 (0.26 to 0.28)	0.28 (0.27 to 0.28)	–0.01 (–0.02 to 0.002)
**EQ-5D VAS**			
Baseline	60.9 (57.4 to 62.8)	61.0 (58.5 to 63.6)	
Baseline to 6 months	9.1 (8.2 to 10.1)	8.0 (7.5 to 8.6)	1.1 (0.1 to 2.2) [a]
Baseline to 12 months	10.1 (9.0 to 11.0)	8.2 (7.6 to 8.8)	1.8 (0.7 to 3.0) [a]

[a] Significant difference.

CI: 95% confidence interval, NRS: Numeric Rating Scale (NRS), EQ-5D: EuroQol 5D, VAS: Visual Analog Scale.

Table S4. Mean travel distance in kilometers (km) to a hospital in the Netherlands for total hip, total knee, and unicompartmental knee arthroplasty patients stratified by private and public hospitals per province

		THA		TKA		UKA	
Province	Hospital type	Mean dist. km	Mean difference (CI)	Mean dist. km	Mean difference (CI)	Mean dist. km	Mean difference (CI)
1	Private	45	31 (30.8 to 31.9) [a]	39	28 (27.2 to 28.1) [a]	34	21 (20.0 to 22.5) [a]
	Public	14		11		13	
2	Private	39	23 (22.2 to 23.2) [a]	40	22 (21.4 to 22.5) [a]	40	23 (21.8 to 24.0) [a]
	Public	16		18		17	
3	Private	35	22 (21.3 to 22.2) [a]	36	23 (22.2 to 23.4) [a]	37	21 (19.4 to 22.1) [a]
	Public	13		13		16	
4	Private	74	50 (47.7 to 51.4) [a]	74	49 (47.1 to 50.0) [a]	59	29 (25.2 to 32.3) [a]
	Public	25		25		30	

5	Private	n.a.	n.a.	n.a.	n.a.	n.a.	n.a.
	Public	18		17		18	
6	Private	63	47 (45.3 to 47.8) [a]	60	43 (41.7 to 44.8) [a]	72	51 (45.6 to 56.9) [a]
	Public	17		17		20	
7	Private	11	–3 (–3.7 to –1.8) [a]	10	–4 (–5.3 to –3.2) [a]	12	–3 (–5.3 to –1.4) [a]
	Public	14		14		15	
8	Private	n.a.	n.a.	n.a.	n.a.	n.a.	n.a.
	Public	21		20		21	
9	Private	n.a.	n.a.	n.a.	n.a.	n.a.	n.a.
	Public	20		19		22	
10	Private	n.a.	n.a.	n.a.	n.a.	n.a.	n.a.
	Public	21		21		26	
11	Private	n.a.	n.a.	n.a.	n.a.	n.a.	n.a.
	Public	22		22		23	
12	Private	47	33 (24.2 to 40.9) [a]	63	49 (47.4 to 50.9) [a]	66	55 (51.3 to 57.7) [a]
	Public	14		14		11	
Total	Private	41	24 (23.9 to 24.4) [a]	41	24 (24.0 to 24.6) [a]	38	19 (18.7 to 20.0) [a]
	Public	17		17		19	

[a] Significant difference.

CI: 95% confidence interval, n.a.: not applicable.

THA	TKA	UKA

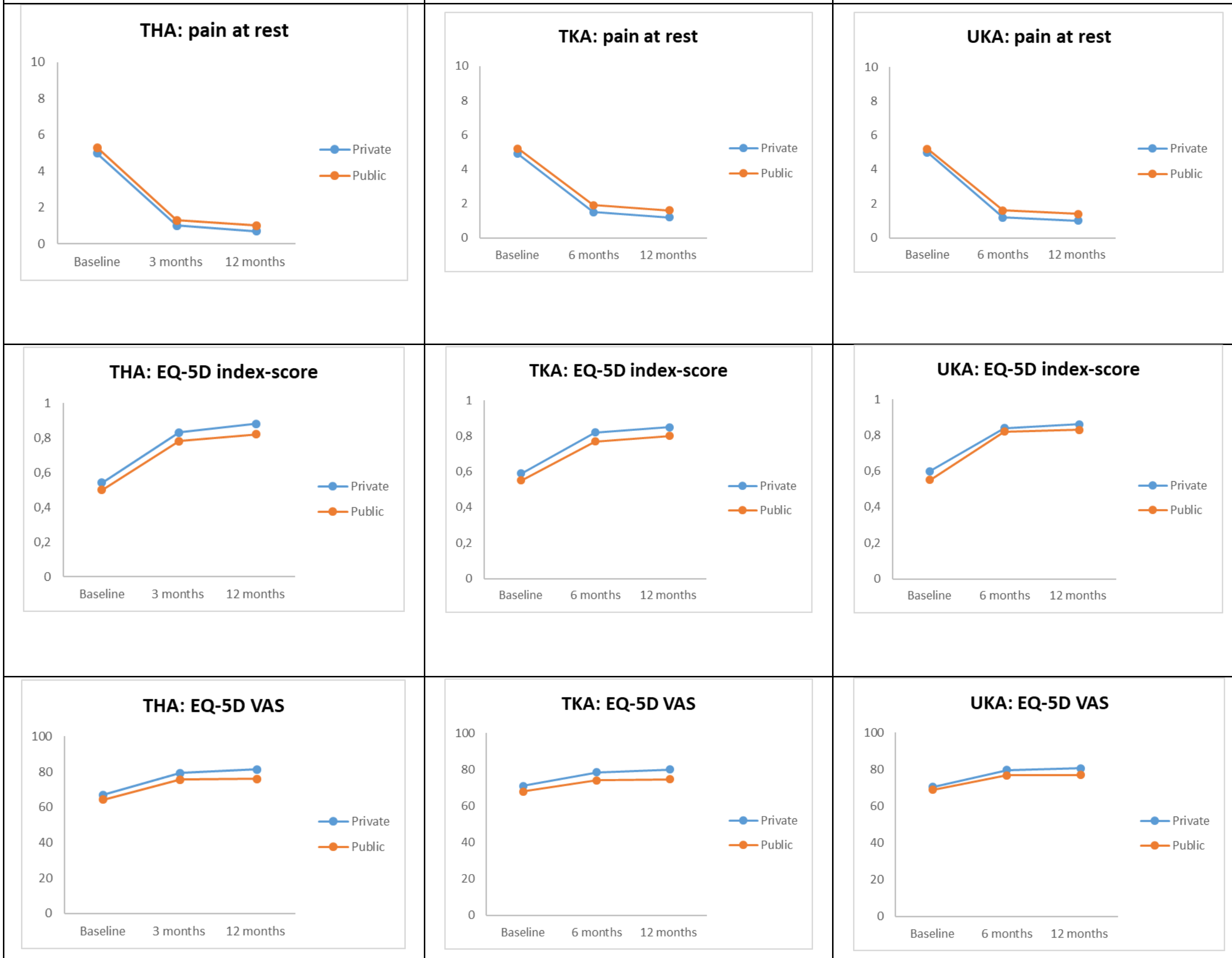

Figure S3. Primary total hip arthroplasties (THA), total knee arthroplasties (TKA), and unicondylar knee arthroplasties (UKA) for Numeric Rating Scale (NRS) pain at rest, EQ-5D index score, and EQ-5D VAS preoperatively, 3- or 6-, and 12- months postoperatively for different hospital types.

NRS: Numeric Rating Scale (NRS), EQ-5D: EuroQol 5D, VAS: Visual Analog Scale.
